# Options for Optimal Coverage of Free C-Section Services for Poor Mothers in Indian State of Gujarat: Location Allocation Analysis Using GIS

**DOI:** 10.1371/journal.pone.0137122

**Published:** 2015-09-02

**Authors:** Kranti Suresh Vora, Sandul Yasobant, Raja Sengupta, Ayesha De Costa, Ashish Upadhyay, Dileep V. Mavalankar

**Affiliations:** 1 Indian Institute of Public Health Gandhinagar, Ahmedabad, Gujarat, India; 2 Department of Geography, McGill University, Montreal, Quebec, Canada; 3 Department of Public Health Sciences, Karolinska Institutet, Stockholm, Sweden; Örebro University, SWEDEN

## Abstract

**Background:**

Gujarat, a western state of India, has seen a steep rise in the proportion of institutional deliveries over the last decade. However, there has been a limited access to cesarean section (C-Section) deliveries for complicated obstetric cases especially for poor rural women. C-section is a lifesaving intervention that can prevent both maternal and perinatal mortality. Poor women bear a disproportionate burden of maternal mortality, and lack of access to C-section, especially for these women, is an important contributor for high maternal and perinatal mortality in resource limited settings. To improve access for this underserved population in the context of inadequate public provision of emergency obstetric services, the state government of Gujarat initiated a public private partnership program called “Chiranjeevi Yojana” (CY) in 2005 to increase the number of facilities providing free C-section services. This study aimed to analyze the current availability of these services in three districts of Gujarat and to identify the best locations for additional service centres to optimize access to free C-section services using Geographic Information System technology.

**Methodology:**

Supply and demand for obstetric care were calculated using secondary data from sources such as Census and primary data from cross-sectional facility survey. The study is unique in using primary data from facilities, which was collected in 2012–13. Information on obstetric beds and functionality of facilities to calculate supply was collected using pretested questionnaire by trained researchers after obtaining written consent from the participating facilities. Census data of population and birth rates for the study districts was used for demand calculations. Location-allocation model of ArcGIS 10 was used for analyses.

**Results:**

Currently, about 50 to 84% of populations in all three study districts have access to free C-section facilities within a 20km radius. The model suggests that about 80–96% of the population can be covered for free C-section services with addition of 4–6 centres in critical but underserved regions. It was also suggested that upgrading of public sector facilities with minimal investment can improve the services.

**Conclusion:**

This study highlights utility of Geographic Information System technology for planning service centres to optimize access to vital lifesaving procedure such as C-section. Although the location allocation methodology has been available for decades, it has been used sparsely by public health professionals. This paper makes an important contribution to the literature for use of the method for planning in resource limited settings.

## Background

Cesarean Section (C-section) is a lifesaving intervention that can prevent both maternal and perinatal mortality. The population-based C-section rate (number of C-sections performed as a proportion of all births) is often used as an indicator of access to life-saving obstetric care. Lack of access to C-section, particularly for poor women who bear a disproportionate burden of maternal mortality, is an important contributor to high maternal (and perinatal) mortality in resource-limited settings [1].

Gujarat, a state in Western India with about 60 million people, has a relatively high gross domestic product (GDP) compared to other Indian states. It has seen strong economic growth of 10.5% in 2011–12, significantly higher than the national average of 8.5% [2]. Unfortunately, the health indicators in Gujarat have been lagging despite the economic growth of the state in the past decade [3,4]. For example, national maternal mortality ratio (MMR) fell from 254 to 178 deaths / 100,000 live births between 2004–06 and 2010–12. The corresponding decline in Gujarat has been modest; from 160 to 112 [5,6,7]. Given this slow progress, Gujarat may not reach the Millennium Development Goal-5 (MDG-5) of 75% reduction in MMR by 2015 [8]. Reduction in MMR is slow, despite a significant increase (32%) in institutional deliveries from 58% in 2004–06 to 90% in 2010–12 [9,10,11]. One of the major reasons for relatively high MMR in presence of high proportions of institutional deliveries could be lack of access to lifesaving procedures such as C-section for complicated obstetric cases [1, 9]. District Level Household Survey-3 (2006–07) data confirms that the proportion of C- section in Gujarat is low at 5%, compared to national proportion of 9%. In the same data, C-section rates for poor Gujarati women are even lower at 2% which has serious implications for MMR reduction [9,12]. This could be due to limited numbers of facilities providing free C-section services in rural areas.

A key reason for limited availability of free C-section in rural areas is the inability of the public sector in Gujarat to recruit and retain obstetricians at sub-district level. As per last available data (2012), there was a 97% shortfall in obstetricians in the public sector in Gujarat [13]. Given this severe shortage of obstetricians in the public sector, and a significant presence of private sector obstetricians at the sub district level, the government of Gujarat introduced “Chiranjeevi Yojana” (CY) in 2005. CY is a public private partnership where a private obstetrician is contracted to provide free delivery care for Below Poverty Line (BPL) and Schedule Tribe (ST) mothers. As provision of C-section is concentrated in the private sector, poor women face financial barriers to using these services. Under the CY program, participating private obstetricians (whose facilities must be capable of performing a C-section) are paid a fixed sum per 100 deliveries irrespective of type of delivery conducted. The sum is based on an average cost per delivery assuming 85 normal deliveries and 15 complicated deliveries, including 7 needing cesareans per 100 deliveries. Since its implementation, about 1 million births have happened under CY and the proportion C-section births among poor mothers in the state have improved from 2% to 6% under the program [11,14,15,16,17].

Though the CY has increased the availability of free C-Section services across the state, there are certain areas where such services are not available or are inadequate. Further, there is a need to identify areas with poor physical access and ascertain options to optimize coverage of free C-section services within the context of CY implementation with the use of novel Geographic Information System (GIS) methodologies. This study assessed the current availability of free C- section services in the public sector and private sector under the CY program in three districts of Gujarat, and then identifies the location of additional free C-section service centers that could optimize the availability of services. No studies have examined current availability of free C-section services including CY providers. The broader purpose of this study was to demonstrate use of GIS methodologies such as location allocation for analysis and evaluation of policy/ program options for optimal coverage of health services.

GIS (along with its set of tools for spatial analysis) is useful to study the distribution and reorganization of health care facilities, including how the delivery of healthcare can be improved by better location of the facilities through planning and evaluation [18]. The normative location-allocation model within GIS is a spatial analytical technique which is used in this study to allocate the demand for services (e.g., obstetric beds) from several locations (e.g., villages) to central supply centers offering C-section services that can meet this demand [19]. This model has been used to measure geographical availability and coverage of population globally, both in developed countries and in the developing countries [20,21,22,23,24,25]. These studies show that location-allocation models are useful to analyze availability and distribution of healthcare facilities and assist with decision-making via what-if? scenarios for policy makers and program managers.

C-section is vital for reduction of maternal mortality and an important aspect of universal health care. Currently, there is limited use of GIS in developing countries for service delivery and dearth of literature to build an evidence base for routine use of GIS for program planning and policy making. Use of GIS offers different options to program planners and policy makers to optimize limited resources hence the study is important for global setting. Irrespective of geographic scope of the study, it is relevant to the global audience as this is the first paper to use GIS to not only examine availability of lifesaving services such as C-section but also use location-allocation modeling for optimizing the availability.

## Methodology

### Study setting

**Fig 1:**
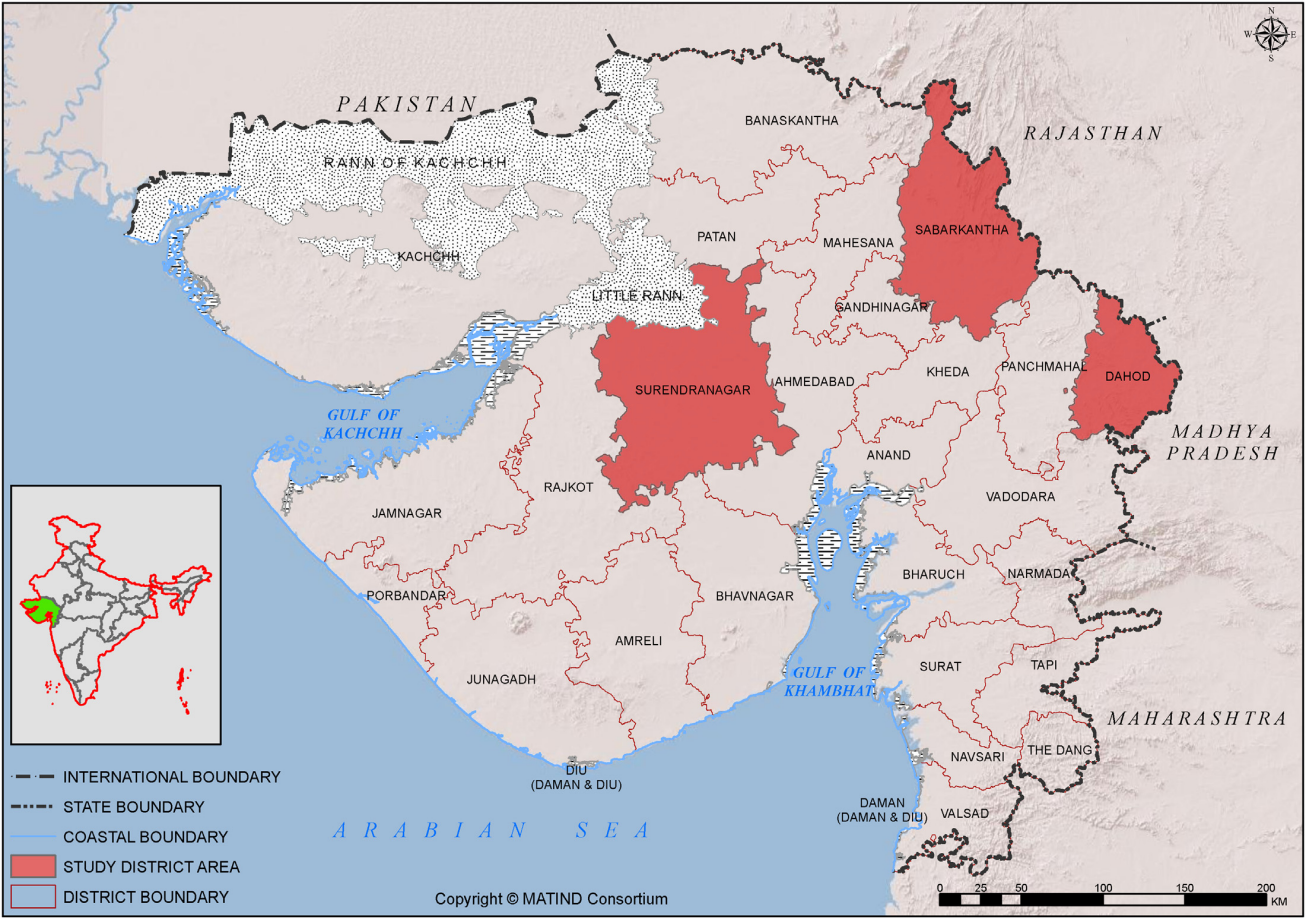
MATIND study districts of Gujarat, India.

Gujarat state is composed of 26 districts, the average population of a district being 2 million. Districts are further divided into 10–20 blocks (sub-districts) that have populations of approximately 100,000 to 200,000. Availability of free C-Section services in three heterogeneous districts from the western, central and eastern belts of the state; Sabarkantha, Surendranagar and Dahod were selected for this study (Fig 1) [26]. These districts have varying human development indices and different population compositions i.e. varying proportions of scheduled castes and tribes and populations living below the poverty line [27].

#### Data collection

Cross-sectional facility survey was done from June 2012 to April 2013. An initial list of all public and private health facilities that routinely provided intrapartum care was obtained from the district public health officials. These facilities and local pharmacies were approached to identify remaining private facilities that were not on the initial listing. The number of deliveries performed in the previous three months for each of the identified facilities was ascertained. All facilities conducting more than 30 deliveries in the last 3 months were recruited in the study. 129 facilities were surveyed; 37 accredited CY private, 36 public sector and 56 non-CY accredited private facilities. Prior permission was obtained for data collection from the appropriate authority. Data on the characteristics; obstetric bed strength and location of all study facilities including information on performance of C-section and CY participation in case of private facilities were collected through a pre-tested questionnaire in vernacular language (Gujarati) administered by trained qualified research staff. The research staff visited the facility to collect information on location of the facility using Global Positioning Systems (GPS) and observed the number of beds available for obstetric services. The research staff was in the facility for 5 days and observed if C-sections are being performed or not. All the members of research staff were trained nursing professionals. Verification of CY participation was done by obtaining list from district authorities implementing the program.

#### Ethics

Ethical approval was taken from Institutional Ethics Committee of Indian Institute of Public Health Gandhinagar for the primary survey. Written consent was obtained from the participating facilities. No individual level data is used for this analysis and facility data was anonymized. Secondary data such as Census is available in the public domain.

### Data sources

Spatially-explicit data were collected from a variety of sources for the three study districts (Sabarkantha, Surendranagar and Dahod, as shown in Fig 1) of Gujarat. Village level population data was obtained from Census of India (2011). The Indian Census is a major source of information on population characteristics which is conducted every 10 years by the office of The Registrar General & Census Commissioner, Government of India. Census 2011 is the 15^th^ National Census of the Country [28].

1. Administrative Atlas of the Census of India (2011) provided village, block and district boundaries for the base map for three study districts and populations
2. Road network were digitized from Survey of India Topo sheets (1975) [29] and updated by manual on-screen digitizing using Google Earth imagery (2014). Both these sources were combined to triangulate the road network information and open series map was also used to augment rather old Topo sheets information.
3. Cross-sectional survey of facilities of the study districts conducted between June 2012 and April 2013 to collect information on facility characteristics such as obstetric beds available and location [30].

#### Base map preparation

We used ArcGIS to first digitize Topo sheets and open series maps into ArcGIS’s shape file format from the paper based hard copy format. The information digitized from these paper maps included village settlement locations and road networks required to operationalize location-allocation method. For preparation of these thematic maps in digital format, Acquisition of topo sheets 1975 and Open Series maps 2001 from Survey of India as hard copies covering study area base on Survey of India (SOI) Index Grid 1:50000 scales are scanned to make them as softcopy; this softcopy is in “Tiff” or “Jpg” format. Toposheets, which are in “tiff” & “Jpg” format are imported in to ERDAS software to make them as ERDAS native format. The geometric correction of toposheet has been done in “geometric latitude/longitude” projection and “Modified Everest India Nepal” datum by placing 4 Ground Control Points on 4 corners of a topo sheet by entering the corresponding latitude/longitude values, which are geo-referenced and mosaicked to form a continuous terrain of the study area. By using ArcGIS 10 software, these topo sheets are digitized theme wise using point, line, and polygon feature. Thematic maps have been developed from SOI Topo sheet and Google satellite image covering the selected district were geo referenced. The individual layers like Roads, Railway, Major River and Settlement / Village locations maps using ARCGIS 10 Software to fix 1:50,000 scale. In the ArcGIS software without moving the scale, visible topo sheet features in 1:50,000 scales have been digitized and additional new feature like roads and railway digitized from On line Google Imagery and Maps, Open Street Map and visualization for Shaded Relief (Elevation) and Terrain map.

However, both these sources had some of the road information missing since they were rather old maps and did not include recently constructed roads and unpaved roads. Using satellite images from the Google Earth, missing road network information were added to complete the road network in the base map. It was challenging to combine datasets from three different sources especially because both Topo sheets and open source maps are paper maps at a very small scale whereas Google Earth is a digital map that provides very high resolution. The topology errors resulting from digitization of paper maps were fixed after visually inspecting and validating the information using the Google Earth maps.

#### Mapping of healthcare facilities

Both the public and private healthcare facilities irrespective of participation in CY were mapped through global positioning system (GPS) devices. The GPS locations were collected at facility main entrance area or relatively open area, away from tall buildings, and out from under tree canopy in order to receive adequate satellite signal strength. Each facility location data saved in the GPS receiver’s memory as well as on a survey tool designed for Facility listing in paper form to avoid errors in any point of time. The saved GPS data is called a waypoint and each waypoint has a unique ID. When a waypoint is saved, the GPS receiver assigns it a default name. The geographic coordinates of these facility sites were also recorded using GARMIN GPS in Degree Minute Second (DMS) format and Altitude (elevation) in meters. After the GPS data has been downloaded into a GIS shape file, the data verification and validation process done for each geographic area. The GPS locations were then geocoded on base-map as discussed above.

### Calculating demand for C-section services (obstetric bed days required)

The demand for C-section services was calculated in two main steps: calculating births per day for each village and calculating “obstetric bed days” required for each village.

Demographic data from Census 2011 i.e. Crude Birth Rate (CBR) at the block level (i.e., an administrative unit of 90–100 villages) and the population of a village were used to generate the number of births per year for each village (X). Number of births per day (Y) was calculated for each village by dividing X by 365.

$$X\;=\;\left\{\frac{CBR(Block\;Level)}{1000}\right\}*Village\;Population$$

Y = (X)/365

To ensure that the calculations made are as realistic as possible, World Health Organization (WHO) assumption of 15% of births being complicated has been used [31]. Current federal government norms in India mandate at least 2 days of stay for normal deliveries and MATIND facility survey showed on an average 5-day stay for C-section deliveries. The final calculations of “obstetric bed days” takes into account this breakup of normal and complicated deliveries and differential hospital stay for the same [32]. All complicated deliveries have been allocated 5 days of hospital stay to avoid underestimation of demand.

Obstetric Bed Days required (Demand)={(Y*0.85*2)+(Y*0.15*5)}

The demand was estimated in this analysis as “obstetrics bed days” required per 1000 populations.

### Calculating supply of free C-section services (obstetric bed days available)

WHO considers hospital beds to be the most feasible indicator of availability of in-patient services [33]. The supply of obstetric bed days available at each center was calculated by multiplying the obstetric bed strength (number of obstetric beds available in the facility) by 365 days.

Supply centers were defined as facilities that are currently providing free C-section services-these could be either public sector or CY private facilities. In the case of two competing supply centre that were located close to each other, the GIS software would choose one with highest capacity and ignore the other one as that supply center by itself is capable of catering to the demand. This is a limitation of this analysis as both facilities together could provide adequate care.

Candidate centers were defined as either (a) private providers that do not provide free C-section services currently but can be contracted under CY, or (b) all the public sector providers with basic obstetric care centers but lacking C-section services. These are potential facilities that could be operationalized to provide free C-section services.

### Calculating capacity of each center to provide free C-section services

First each center was designated as a supply center to all villages within a 20 km radius of the center. 20 km radius is considered to the reasonable catchment area as it is an approximate distance covered by ambulances in 1 hour along Indian sub-district roads [34]. As per United Nations (UN) indicators for monitoring Emergency Obstetric Care (EmOC) services, 2 hours is the minimum duration between onset of major obstetric complications and maternal mortality. Factoring in the time needed for decision-making and arranging for the transport, 1 hour travel time is considered to be an acceptable boundary for catchment area in this study [35]. The distances measured between demand (villages) and supply (facilities) locations were along a road network. The demand for obstetric bed days in catchment area of a center was assessed as a sum of demand of all villages served by that centre. Capacity [36] of each center was calculated based on the difference between supply (available obstetric bed days) and demand (Obstetric bed days required per 1000 populations). If the supply was less than demand then the center was categorized as “under capacity” while if the supply was enough or more than demand then the center was categorized as “over capacity”.

### GIS analysis—location allocation model

To minimize the total distance between villages and nearest supply centers, a location-allocation model (p-median model) attempts to select the future supply centers, from a set of candidate centers [37]. This ‘minimum distance’ is additionally weighted according to the characteristic of the demand location irrespective of distance such as demand sites further away from a supply site with a larger population can be given a higher weight than closer demand sites with smaller population, if the weighting criteria is ‘demand’. A widely used variant of the p-median model of ArcGIS 10 [38] that maximizes service coverage while minimizing distance weighted by demand was utilized in this study [39,40,41,42]. Moreover, capacity constraints of a supply center were considered along with travel distance to such a center from the demand node [43].

The current scenario of coverage provided by the existing supply centers were analyzed through a run of the maximum coverage model with distance and capacity constraint. The potential for improvement in coverage was analyzed by incrementally allowing the model to select additional potential locations by adding candidate centres one by one and observing change in the proportion of coverage. The final set of supply centers was proposed when it was observed that adding new centers yielded no further significant improvements in coverage.

## Results

Sabarkantha, Dahod and Surendranagar had respectively 23, 8 and 18 supply centers that currently provide free C-section services to the women residing there. Table 1 shows the summary of current coverage and optimal coverage of free C-section by supply centers. In the current scenario, supply centers cover 84% of the population in Sabarkantha, 65% in Dahod and the least 48% in Surendranagar. Running the location-allocation model shows that there is a potential of substantial increase in the percentage of population covered in these districts within 20 km through the recruitment of a few candidate centers and ensuring these provide free C-section services. In Sabarkantha district, an additional 5 centers increase the population coverage by 12%, to total of 96%, in Dahod, 4 additional centers will lead to a 31% increase in coverage. In Surendranagar district adding 6 centers increases the coverage by 32% and yields an overall coverage of 80%. These findings indicate difference among districts regarding availability and geographic location of candidate centers leading to variation in optimal coverage and number of facilities required.

**Table 1:** Summarizes current and potential optimal coverage for all three Districts.

*District*	*Total Population (In Million)*	*No*. *of Current Supply Centres*	*Current Coverage of Population* * *(In Million)*	*% of Population Covered Currently*	*No*. *of Candidate Centres*	*Total Population Covered after Allocation* * *(In Million)*	*% of Population Covered after Allocation*	*% Point Increase in Coverage*
***Dahod***	2.12	8	1.38	65	4	2.05	96	32
***Sabarkantha***	2.42	23	2.04	84	5	2.34	96	13
***Surendranagar***	1.75	18	0.84	48	6	1.40	80	32

*Based on villages falling into a 20 km radius catchment area

**Fig 2:**
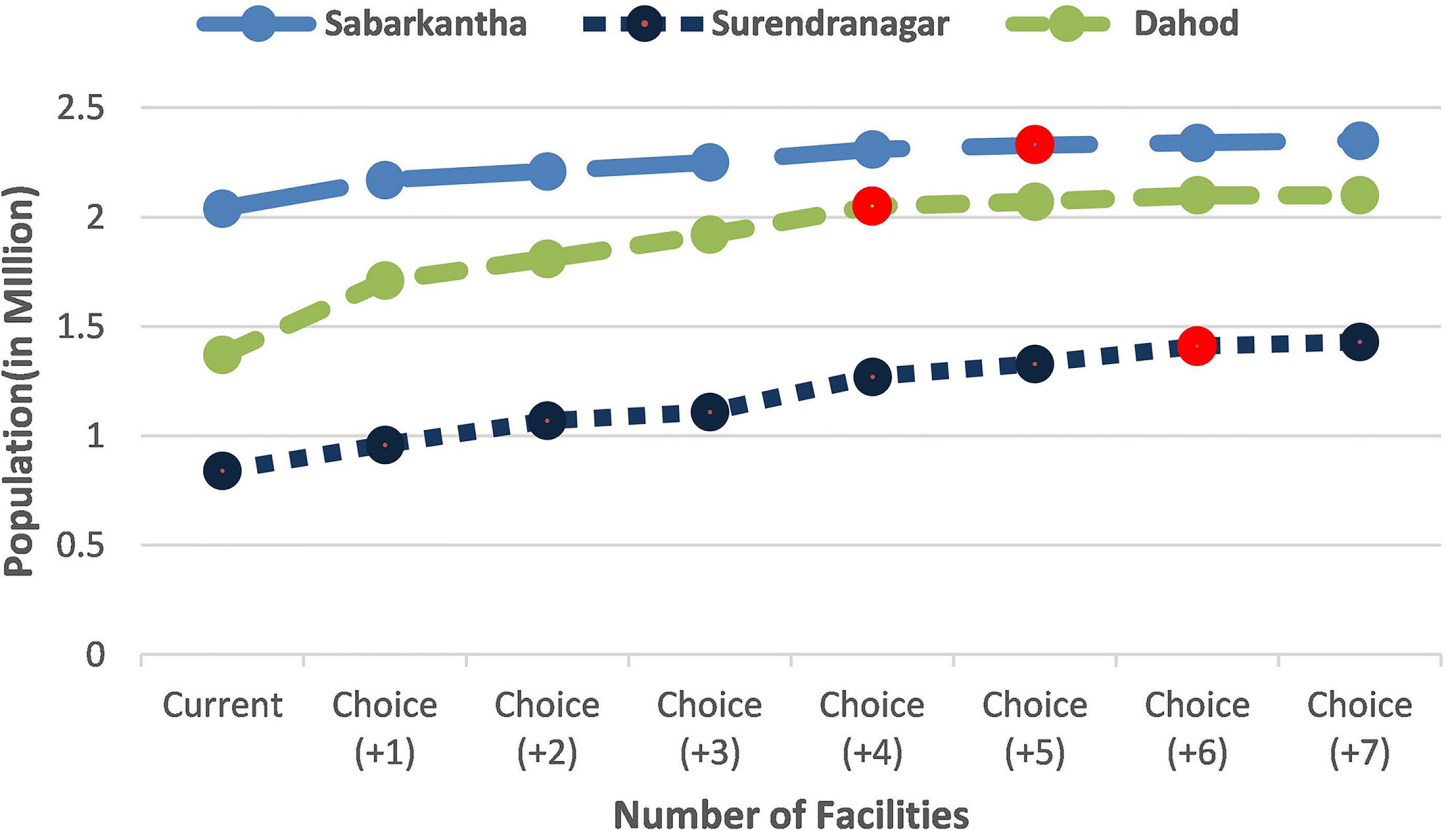
Choice of candidate facilities and resultant increase in population coverage for free C-section services.

As seen in the Fig 2, adding candidate centers one by one, the population coverage increased in all districts. The level of increase differed by district with the highest increase seen in Dahod and least in Surendranagar after adding one candidate center to current supply centers; Note that after the first addition, the level of increase evens out. As explained in the methods section (model description), the model stopped adding candidate centers when there was no substantial increase in the population coverage which is denoted in the figure.

**Fig 3:**
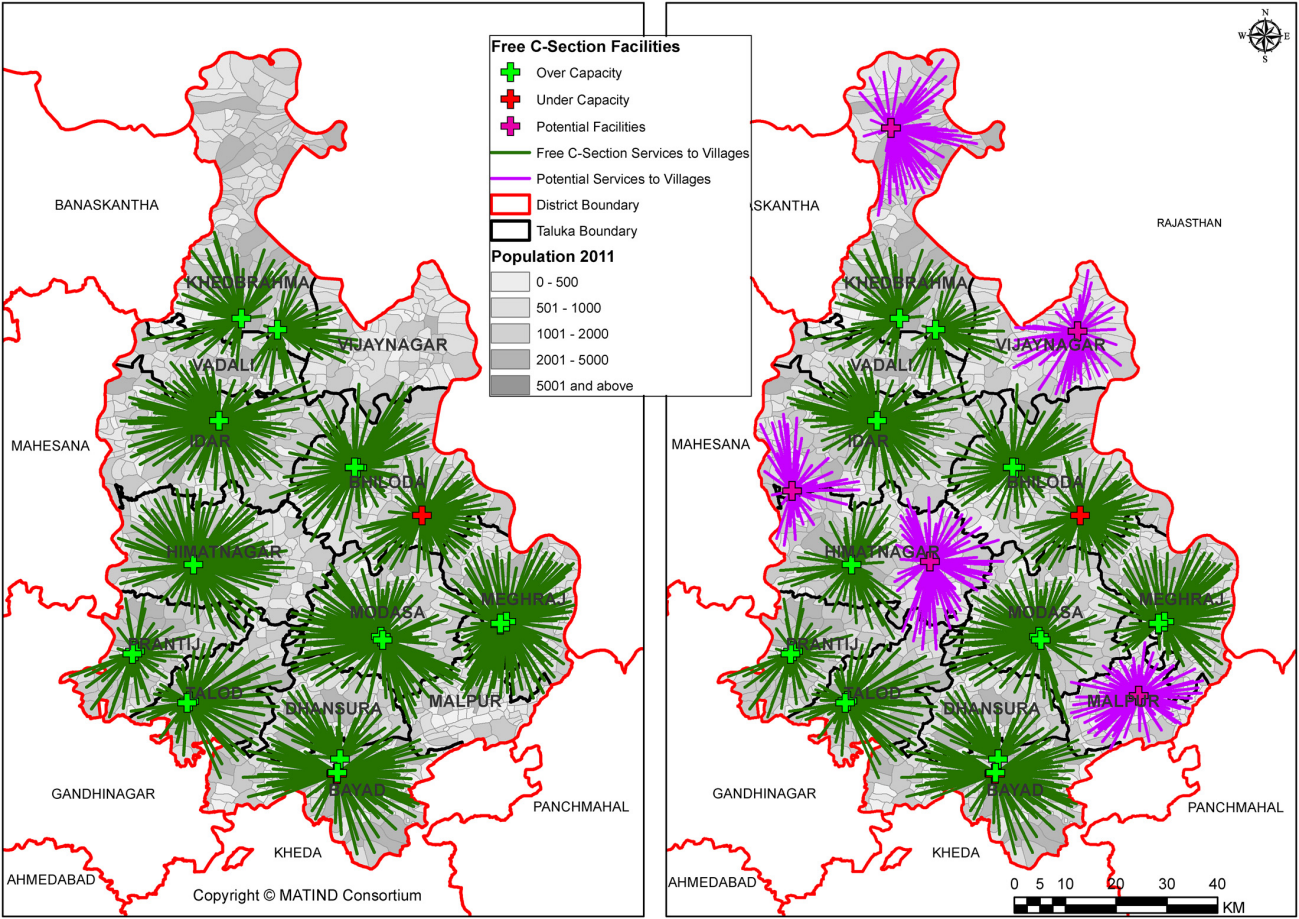
(a & b): Current and Potential coverage to services in Sabarkantha.

As seen in the maps below in Fig 3a & 3b, out of 23 current supply centers, 2 are under capacity (i.e. do not have adequate obstetric beds) and 21 are overcapacity (i.e. have adequate or more obstetric beds available) in Sabarkantha district which indicates oversupply of services in this district. It should be noted that despite this oversupply, there are areas where services are not available (Northern part). With addition of 5 candidate centers, the number of under capacity centers reduces to 1 and the areas with absence of services are also covered. While in Dahod district, all supply centers are under capacity.

**Fig 4:**
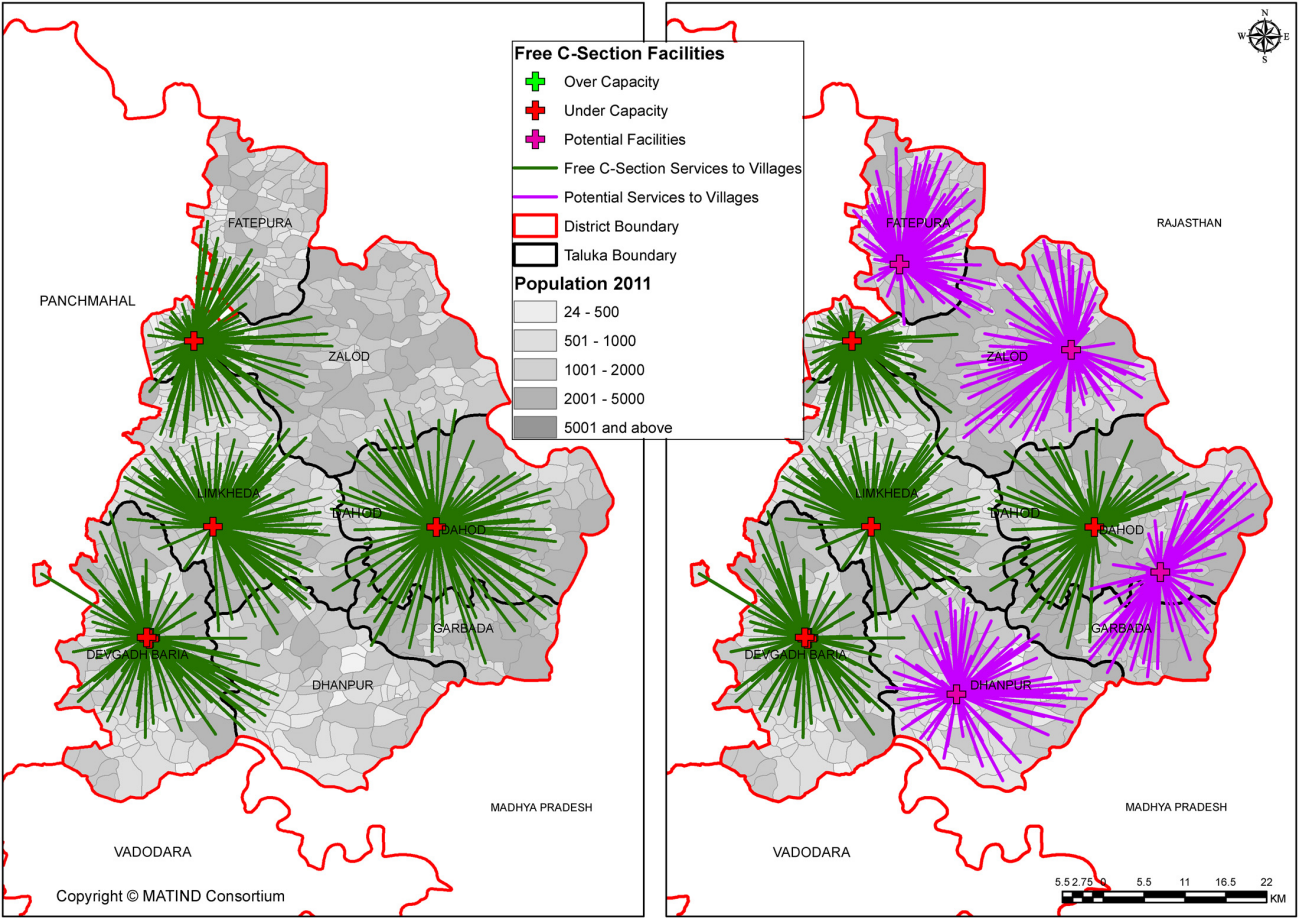
(a & b): Current and Potential coverage to services in Dahod.

As seen in Fig 4a & 4b, after addition of 4 candidate centers, the number of under capacity centers remains the same in Dahod which indicates gross under provision of free C-section services in this district. It is worth noting that the population coverage is 96% in Dahod after addition; which mean there is a need to increase the capacity of supply and candidate centers.

**Fig 5:**
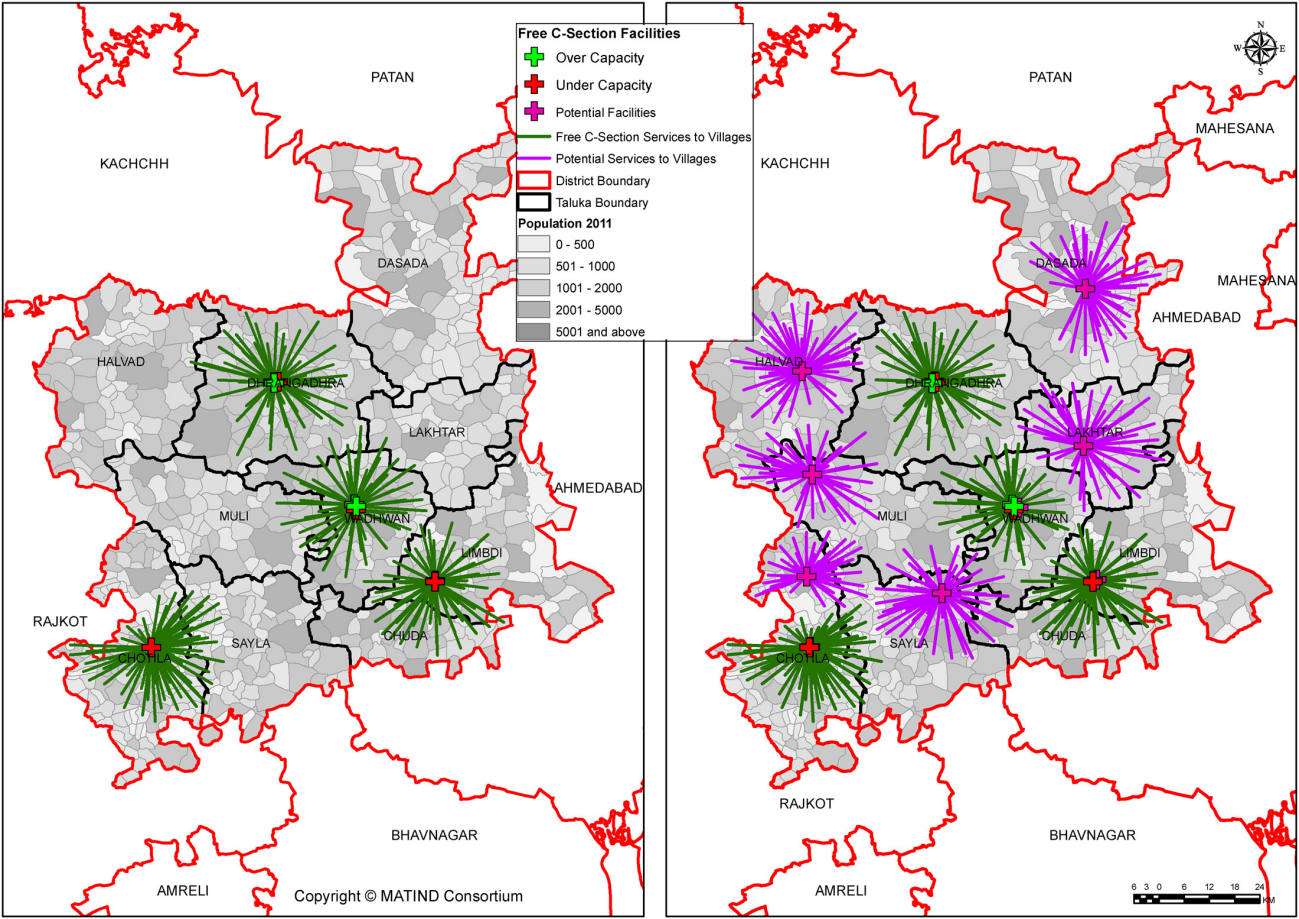
(a & b): Current and Potential coverage to services in Surendranagar.

Surendranagar district had low coverage to begin with at 48% with 18 supply centers; Out of these, 4 are under capacity. Addition of 6 candidate centers increased the coverage only up to 80% and the number of under capacity centers did not change as seen in Fig 5a & 5b. Also, availability of free C-section services within certain areas in the northeastern part which are adjacent to a desert area (geographically difficult area) remains limited despite addition of candidate center. This indicates that, there is not only a need to add more centers but also to increase significantly the capacity of existing centers to provide C-sections.

Results indicate that all three study districts differ in level of current coverage, geographic location and distribution and capacity of supply centers. The location-allocation model suggest that with addition of candidate centers, the coverage of free C-section services can be improve significantly but the capacity of candidate and supply centers need to be increased in Dahod and Surendranagar.

Table 2 shows the current capacity or ability of the facilities to provide services and the change seen after additional facilities. It should be noted that except for Sabarkantha district, in other two districts the capacity did not change even after additional facilities. This signifies the dearth of obstetric beds in facilities providing EmOC in study districts.

**Table 2:** Change in Capacity of Facilities pre & post allocation period.

*District*	*No*. *of Current Supply Centres*	*No*. *of current Under capacity centres*	*No*. *of current Over capacity centres*	*No*. *of Candidate Centres*	*No*. *of Under capacity Centres after allocation*	*No*. *of Over capacity Centres after allocation*
***Dahod***	8	7	1	4	7	1
***Sabarkantha***	23	2	21	5	1	22
***Surendranagar***	18	4	14	6	4	14

### Limitations

This model is limited to the district boundaries, and there is no crossover across district or state boundaries. As three districts selected, share major proportion of their boundaries with areas that have under provision of free C-section services such as Kutch district for Surendranagar which is a desert area with limited availability of services. As a result there is no significant crossover from the study districts to areas outside district boundaries although the opposite could be true. A second assumption was ignoring existing supply center for allocation part of the model if it has lower capacity than an adjacent one seems artificial. This may lead to oversupply of services to the underprivileged section of the society, which is beneficial not harmful. For calculating supply, all the facilities providing free C-section services are taken into consideration. Despite these assumptions the model provides a reasonable estimation of demand and supply of free C-section services and potential locations to optimize the coverage.

## Discussion

Study findings suggest that the current population coverage to free C-section services within 20 km in the study districts by public sector and private sector (CY facilities) is limited (48–80%). Even after additional facilities, there are proportions of populations (4–20%) that have limited availability within 20 km. Moreover if the numbers of obstetric beds in current and potential facilities are taken into consideration, the ability of providing free C-section services is less than required. This finding indicates the need to improve bed strength of existing and additional facilities to ensure adequate supply of EmOC services. This is the first study to include number of beds (capacity of the facility to provide services) for demand and supply analysis. This gives a realistic picture of availability of services and not just the geographic location of the facility.

Within the model, potential sites prioritized for optimizing free care were mainly secondary level public sector health facilities that have majority of required resources in place. Literature suggests that low obstetric performance in public sector facilities in India are mostly due to human resources issues such as scarcity of obstetrician, lack of skills to manage obstetric complications [44,45] The rationale for the selection of potential facilities was to optimize coverage with minimal investment which could be just appointing an obstetrician in a vacant post—within the public sector. Currently, public sector facilities are underutilized in Gujarat while 60% of institutional deliveries take place in the private sector [11]. Upgrading secondary level public sector facilities will improve utilization of existing resources and improve availability of free C-section services for poor mothers. The Indian government has implemented innovative strategies such as training general doctors for C-section and anesthesia in collaboration with professional organizations (replace India case study) [46].

Table 3 summarizes analysis findings and describes possible policy options. Policy option (not shown in the model) in the context of Gujarat is contracting current non-CY private facilities to join CY. In areas where none of these are available, establishment of a new public sector center or incentivizing a fresh graduate to start private practice are some other policy options that can be explored. As indicated in Table 3, in sparsely populated areas, the lower level facilities can be connected to higher level facilities by efficient referral linkages to ensure coverage although in this case the distance might be more than 20 km. An important finding of the study is that even after ensuring optimal access, some of the facilities do not have adequate numbers of beds to cater to the demand. Upgrading the public sector facilities by adding additional resources required to increase the bed capacity or enrolling more private providers in CY can improve the situation.

**Table 3:** Summary of Key findings from Location-Allocation Model Analysis with policy options.

District	Key findings from the Model Analysis	Possible Policy options
Dahod	• Current population coverage 65%	• Increase the obstetric bed capacity of existing and candidate centres
	• An additional 4 centers increase the population coverage by 31%	• Enroll CY providers in limited availability areas
	• Potential population coverage = 96%	
	• Majority of the facilities do not have sufficient numbers of obstetric beds and this remained unchanged after addition	
Sabarkantha	• Current population coverage = 84%	• Establish public sector facilities or incentivize private sector to provide free services in limited availability area
	• An additional 5 centers increase the population coverage by 12%	• Other option is to establish referral linkages between existing facilities and limited availability villages
	• Potential population coverage = 96%	
	• Out of two facilities that had insufficient obstetric beds only one facility improved after additional facilities	
Surendranagar	• Current population coverage = 48%	• Establish public sector facilities or incentivize private sector to provide free services in limited availability area
	• An additional 6 centers increase the population coverage by only 32	• Other option is to establish referral linkages between existing facilities and limited availability villages
	• Potential population coverage 80%	
	• Majority of the facilities do not have sufficient numbers of obstetric beds and this remained unchanged after addition	

The study findings also suggest that population based UN indicators for availability of C-section are not sensitive enough to capture geographic ground reality hence are not sufficient to indicate ability of the health system to provide optimal EmOC to reduce maternal deaths [47]. In Sabarkantha there are 23 facilities currently providing Comprehensive EmOC (CEmOC) for the population of about 2.4 million [48] which is more than adequate provision of C-section services as per UN indicators (approximately one facility per 100,000 populations versus UN norm of one per 400,000 populations). Yet, there areas in this district that have limited availability and women have to travel far to avail lifesaving obstetric services. Literature suggests the need to include physical availability as an important determinant to skilled birth attendance and there is a need to include geographic indicators in the determining availability of EmOC [49]. There is a need for adding GIS analysis and modeling to get a comprehensive picture of availability of essential services and planning to optimize limited resources. The same method can be used in any resource constraint situation to ensure availability of essential healthcare services with minimal investments to reduce inequity.

### Implication of the study on use of GIS for location of health facilities to optimize services

Location of health services is important to ensure spatial access especially for rural areas. There are few publications detailing use of GIS for location of health facilities for developing countries. A review paper on location allocation models for health services development in developing nations notes that the earliest use of the model was in 1966 in Guatemala and in India a publication in 1979 mentions use of the model for locating social services [50]. One of the important issues related to use of GIS in location is lack of appropriate data such as accurate numbers of births happening to poor women which were not available for the current study. The other important reasons cited in literature for limited use is lack of advocate for use of such technology for planning purposes and expensive GIS software that require experts to analyze and interpret spatial data in the developing world [41, 51,52].

In low and middle income countries where mixed health care market exists and public resources are limited, it is useful to utilize technology to optimize use of available private sector and scarce public resources. As it is possible to reduce cost of healthcare and improve health service availability by redirecting limited resources with use of GIS technology such as location allocation, countries such as India should embrace the same and make them part of routine health care planning process [51]. Current study findings show that access can be maximized for free C-section services with use of location allocation in three backward districts of Indian state of Gujarat.

All the location allocation model studies in developing nations mention constraints of weak health system and limited resources in developing nations as an obstacle to improve access to health care [53],[54]. Current study findings show that with minimal investment in public sector access to free C-section services can be improved significantly in three districts of Indian state of Gujarat. This study also demonstrates the importance of using GIS techniques such as location allocation for optimal coverage of health services in low resource areas and adds to the literature on policy and program options for universal health care.

## Conclusion

The location allocation model is a useful GIS technique used in this study for understanding coverage of free C-section with facility, capacity, and distance constraints. To achieve significant reduction of maternal mortality, it is essential to ensure universal availability to C-section services including free services to poor. Differences in availability of public and private sectors along with geographic diversity among districts highlight the need to use GIS techniques to ensure optimal use of current resources to provide healthcare to all. Location allocation analysis results and the process of selection of potential centers highlighted interesting opportunities, policy options and how GIS methods can be used for planning up gradation of old facilities or establishing new centers to ensure coverage.
